# Immunohistochemical Characterisation of GLUT1, MMP3 and NRF2 in Osteosarcoma

**DOI:** 10.3389/fvets.2021.704598

**Published:** 2021-08-03

**Authors:** Catrin S. Rutland, James M. Cockcroft, Jennifer Lothion-Roy, Anna E. Harris, Jennie N. Jeyapalan, Siobhan Simpson, Aziza Alibhai, Clara Bailey, Alyssa C. Ballard-Reisch, Albert A. Rizvanov, Mark D. Dunning, Simone de Brot, Nigel P. Mongan

**Affiliations:** ^1^School of Veterinary Medicine and Science, Faculty of Medicine and Health Sciences, University of Nottingham, Nottingham, United Kingdom; ^2^Faculty of Medicine and Health Science, Biodiscovery Institute, University of Nottingham, Nottingham, United Kingdom; ^3^Institute of Fundamental Medicine and Biology, Kazan Federal University, Kazan, Russia; ^4^Willows Veterinary Centre and Referral Service, Solihull, United Kingdom; ^5^COMPATH, Institute of Animal Pathology, University of Bern, Bern, Switzerland; ^6^Department of Pharmacology, Weill Cornell Medicine, New York, NY, United States

**Keywords:** osteosarcoma, canine, solute carrier family 2 member 1, matrix metallopeptidase 3, nuclear factor erythroid 2-related factor 2, pathology, cancer identification

## Abstract

Osteosarcoma (OSA) is an aggressive bone malignancy. Unlike many other malignancies, OSA outcomes have not improved in recent decades. One challenge to the development of better diagnostic and therapeutic methods for OSA has been the lack of well characterized experimental model systems. Spontaneous OSA in dogs provides a good model for the disease seen in people and also remains an important veterinary clinical challenge. We recently used RNA sequencing and qRT-PCR to provide a detailed molecular characterization of OSA relative to non-malignant bone in dogs. We identified differential mRNA expression of the solute carrier family 2 member 1 (SLC2A1/GLUT1), matrix metallopeptidase 3 (MMP3) and nuclear factor erythroid 2–related factor 2 (NFE2L2/NRF2) genes in canine OSA tissue in comparison to paired non-tumor tissue. Our present work characterizes protein expression of GLUT1, MMP3 and NRF2 using immunohistochemistry. As these proteins affect key processes such as Wnt activation, heme biosynthesis, glucose transport, understanding their expression and the enriched pathways and gene ontologies enables us to further understand the potential molecular pathways and mechanisms involved in OSA. This study further supports spontaneous OSA in dogs as a model system to inform the development of new methods to diagnose and treat OSA in both dogs and people.

## Introduction

Canine osteosarcoma (OSA) presents a significant veterinary clinical challenge with an estimated incidence rate of between 13.9–27.2/100,000 dogs, considerably higher than the rate in people, 1–3 cases/annum/1,000,000 people (1–4). It shares many clinical and molecular features with human OSA (5–8). The current management of choice for canine OSA is surgery followed by chemotherapy; the one year survival rarely exceeds 45% even for patients receiving treatment (5, 9–14). In contrast to human OSA, canine OSA is most common in middle aged dogs and a degree of heritability has been observed (1, 15, 16). Canine OSA presents a promising model for determining the underlying mechanisms of OSA carcinogenesis and cancer progression, and also provides an opportunity for the development of drugs targeting OSA-specific pathways. Multidrug resistance is a critical limitation to the current success of chemotherapy and, therefore, additional therapeutic approaches are needed that could reduce the metastatic rate and recurrence of OSA (17).

To support the development of more effective therapies, there is a need to understand the underlying mechanisms of OSA etiology and progression. OSA predominantly affects large and giant breed dogs, particularly Irish Wolfhounds, Rottweilers, Deerhounds, St Bernards, and Great Danes, with association made with male dogs and increased height and weight (1, 18). This is comparable to human OSA where male sex and height are associated with higher incidence rates peaking at puberty (6, 19, 20). These findings in humans and canines support the potential role of developmental factors and increased cell proliferation in OSA etiology. Previous studies have implicated ezrin, a membrane cytoskeleton linking protein, in poor prognosis and metastasis (21–24). Evidence also supports a role of epigenetics in the development of OSA, however, this is not yet well understood (25, 26). Well-characterized oncogenes and tumor suppressors, including MYC, EGFR, AKT2, TP53, CDKN2A/B, RB1, BCL2 and PTEN, have also been implicated in canine OSA (15, 25, 27). Karyotypic instability, associated with mutations of *TP53*, is characteristic of OSA (28).

More recently our group identified several genes significantly differentially expressed between canine OSA and non-tumor bone tissue (16). Consistent with the association with bone growth and development, multiple gene ontologies of the differentially expressed genes related to cellular differentiation, morphogenesis, development, cellular proliferation, and metabolism (16). Intracellular signaling, calcium homeostasis and heme synthesis were also implicated. Analysis showed that *MMP3* and *SLC2A1* expression were significantly higher in OSA tissue compared to non-tumor tissue and protein expression in OSA was confirmed by immunohistochemistry. This study expands on the initial analysis (16) by investigating the levels of MMP3, GLUT-1 (protein expressed by *SLC2A1*) and NRF2 (transcription factor encoded by *NFE2L2*), which are known to play a role in human OSA, in an OSA canine cohort.

## Materials and Methods

### Specimen Preparation

All animal tissue work in this study was approved by the Ethics committee at the University of Nottingham School of Veterinary Medicine and Science and complied with national ethics procedures (permission number - UG 20331). Patients were euthanised under normal veterinary practice under circumstances unrelated to research. Diagnosis of OSA was confirmed by a board certified histopathologist.

### Immunohistochemistry and Microscopy

Proteins of interest were identified following gene expression analysis (RNA sequencing), validated by qRT-PCR (16). Immunohistochemistry was performed to show positive protein expression of GLUT1, MMP3 and NRF2. Rottweiler post-mortem OSA tissue (*n* = 15) was obtained from Bridge Pathology, UK in the form of formalin fixed, paraffin embedded OSA tissue. The OSA samples were all excised from Rottweilers, 9/15 female, 5/15 male and 1/15 not specified. The females ages ranged between 7-9 years old and 2/9 were entire, and the males were between 6-10 years old and 3/5 were entire. OSA location was 10/15 appendicular, 3/15 axial, 1/15 mixed appendicular/axial, 1/15 not specified. A range of morphologic types were studied including 10/15 osteoblastic; 3/15 chondroblastic or mixed osteoblastic/chondroblastic; and 2/15 suspected giant cell rich. In addition a range of mitotic activity values [as previously defined (29)] were included: 3/15 value 1; 8/15 value 2; 4/15 value 4. All of these higher mitotic values were observed in females, in addition the two cases with highest mitotic activity overall were females and had large amounts of osteoid. Given the deliberately mixed nature of the OSA samples, statistics were not carried out on location, morphologic type, sex or mitotic activity.

Tissue was post-fixed in 4% paraformaldehyde for 2 hours, dehydrated through an ethanol series, embedded into paraffin blocks, and sectioned at 7 μm. Immunohistochemistry was carried out u Proteins of interest were identified following gene expression analysis (RNA sequencing), validated by qRT-PCR (16). Immunohistochemistry was performed to show positive protein expression of GLUT1, MMP3 and NRF2. Rottweiler post-mortem OSA tissue (*n* = 15) was obtained from Bridge Pathology, UK in the form of formalin fixed, paraffin embedded OSA tissue. OSA samples from a variety of bones including the humerus X2, scapula, femur X3, mandible X2, temporomandibular joint, tibia, maxilla, stifle, carpus and 2 unknown locations were excised from male and female Rottweilers between the ages of 6-11 years old. Tissue was post-fixed in 4% paraformaldehyde for 2 hours, dehydrated through an ethanol series, embedded into paraffin blocks, and sectioned at 7 μm. Immunohistochemistry was carried out using a Leica Novolink Polymer Detection Kit (Leica, Wetzlar, Germany) according to manufacturer's protocols with primary antibodies diluted in fetal calf serum 1:100; anti-SLC2A1(GLUT1) polyclonal unconjugated rabbit antibody (100732-TOB-SIB; Stratech, Ely, UK), anti-MMP3 polyclonal unconjugated rabbit antibody (GTX74514; GeneTex, Irvine, CA, USA), anti-NRF2 (*NFE2L2*) polyclonal unconjugated rabbit antibody (ab31163; Abcam, Cambridge, UK) were used to stain proteins of interest. Microscopy was carried out to confirm positive staining cytoplasmic and/or nuclear staining (Leica, Wetzlar, GermanyUK) and systematic random sampling employed to take photomicrographs for H-scoring. Negative controls received no primary antibody and were incubated in fetal calf serum only. Kidney sections from one of the patients were used as positive controls, as the target markers were known to be expressed in the kidney (30–32).

### H-Scoring

H-scoring, a well-established semi-quantitative technique for protein expression was used to analyse the samples. It is often considered as one of the “gold standards” for immunohistochemistry evaluation (33–35). H-scores were undertaken by one double-blinded researcher who established a scoring definition and then undertook the scoring within a two week period to ensure interpretation consistency. Two additional researchers scored 10% of the samples, chosen randomly to ensure concordance. Staining intensity was designated into scores of 0, 1+, 2+, or 3+ (none, weak, moderate, strong staining signal) for each antibody. The percentage of cells/tissue containing positive staining (to the nearest 5%) of either cytoplasmic and/or nuclear staining were calculated independently for a fixed field of n = 4-5 photomicrographs per sample (*n* = 10-13 OSA samples) for each antibody. H-scores were calculated using the formula: H-score = [1 × (% cells 1+) + 2 × (% cells 2+) + 3 × (% cells 3+)] for both cytoplasmic and nuclear staining separately. H-scores based on the resulting 0–300 scale were calculated for each specimen and each protein. The mean, standard error of the mean, minimum, maximum and range of H-scores were calculated. Data was plotted to demonstrate both score distributions and staining intensities. In addition, representative staining scores (based on these samples only) were created as benchmarks to discuss results. H-scores were also classified as low 0–45, moderate 45-90 or high 90+ average scores in order to describe the overall scores. Statistical analysis between cytoplasmic and nuclear H-scores was conducted using paired *T*-test (SPSS v26). Comparisons between the number of slides with 0, 1, 2, and 3 H-score staining categories in the cytoplasm and nucleus were conducted using chi-square.

Qualitative data was also recorded in order to describe general immunohistochemical staining patterns. Qualitative data was described for both neoplastic areas and, where possible, adjoining areas where no tumor was present. In addition to describing the cell/structure types present and the immunostaining observed, general staining was identified for each sample (diffuse, multifocal, focal), both cytoplasmic and nuclear staining were described (absent, weak, moderate, strong) and the predominant staining location was identified (cytoplasmic, nuclear or equal).

## Results

### GLUT1 H-Score and Expression in OSA

**Figure 1:**
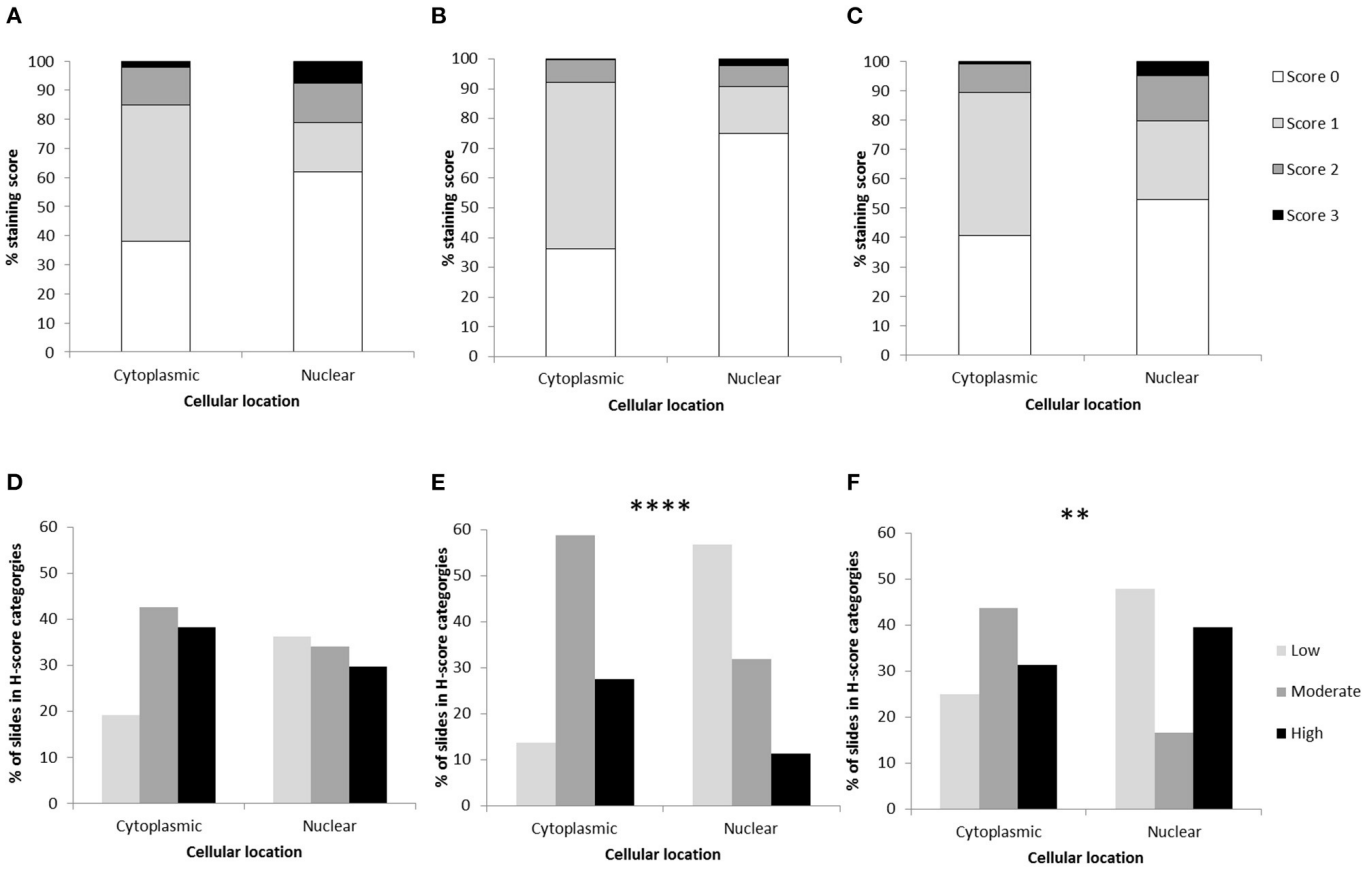
Osteosarcoma H-scores in the cytoplasm and nucleus following immunohistochemical staining. Average H-scores for **(A)** GLUT1, **(B)** MMP3, and **(C)** NRF2. H-score distributions across samples for **(D)** GLUT1 (*P* > 0.05), **(E)** MMP3 (****P < 0.0001), and **(F)** NRF2 (**P = 0.008). Differences between nuclear and cytoplasmic staining were assessed using chi-square.

GLUT1 staining (*n* = 47 sections from 13 patients) showed H-score variations between the different patients, however all specimens showed positive immunostaining. Only 1 of the 13 patients showed both low cytoplasmic and low nuclear average scores (7.7%), while 2 of the 13 patients showed both high cytoplasmic and high nuclear average scores (15.4%). Only one patient had a low average GLUT-1 cytoplasmic score, 7 patients had moderate cytoplasmic scores (58.3%) and 5 had high cytoplasmic scores (38.5%). Nuclear scores showed 4/13 patients with low H scores (30.8%), 4/13 at moderate (30.8%) and 5/13 at high (38.5%), overall these were not significantly different to the cytoplasmic staining scores (*P* > 0.05). The 0-300 cytoplasmic H-scores were slightly higher than the nuclear scores, however the same range for both locations was observed and no significant differences were present (*P* > 0.05, Table 1, Figures 1A,D).

**Table 1:** H-scores from GLUT1, MMP3 and NRF2 immunostained canine OSA specimens showing inter case variation.

		**H-Score**	
**Protein (number of cases)**	**Cellular location**	**Mean ± SEM**	**Range (min-max)**
GLUT1 (*n* = 13)	Cytoplasmic	74.89 ± 11.11	180 (5–185)
	Nuclear	67.15 ± 11.38	180 (5–185)
MMP3 (*n* = 12)	Cytoplasmic	69.88 ± 4.60	95 (25–120)
	Nuclear	38.58 ± 8.61	135 (0–135)
NRF2 (*n* = 10)	Cytoplasmic	71.89 ± 6.42	130 (10–140)
	Nuclear	74.17 ± 13.08	200 (0–200)

*H-Score indicates average scores calculated from several slides for each patient. N = number of cases. Low was classified as 0–45, moderate 45–90 or high was a score of 90+*.

The histopathology general report indicated that all specimens showed diffuse staining distribution, with cytoplasmic staining classified as mostly weak or weak to moderate (Table 2). Nuclear stain intensity ranged from absent to weak – moderate, and the majority of samples showed predominantly cytoplasmic staining, but in some samples the predominant stain was nuclear whereas in others the cytoplasm and nucleus were equally stained (Table 2).

**Table 2:** Overall blinded histopathology assessment for each OSA case.

**Protein**	**Staining distribution**	**Cytoplasmic staining intensity (% of cases)**						**Nuclear staining intensity (% of cases)**						**Predominant staining** **(% of cases)**		
**Protein**	**Diffuse/Multifocal/Focal**	**Absent**	**Absent-Weak**	**Weak**	**Weak-Moderate**	**Moderate**	**Strong**	**Absent**	**Absent-Weak**	**Weak**	**Weak-Moderate**	**Moderate**	**Strong**	**Cytoplasmic**	**Nuclear**	**Equal**
GLUT1	100% diffuse	-	61.54	-	38.46	-	-	23.08	30.77	15.38	30.77	-	-	61.54	23.08	15.38
MMP3	100% diffuse	-	-	75.00	16.67	8.33	-	58.33	16.67	16.67	8.33	-	-	91.66	-	8.33
NRF2	100% diffuse	12.50	12.50	62.50	12.50	-	-	37.50	25.00	37.50	-	-	-	62.50	12.50	25.00

**Figure 2:**
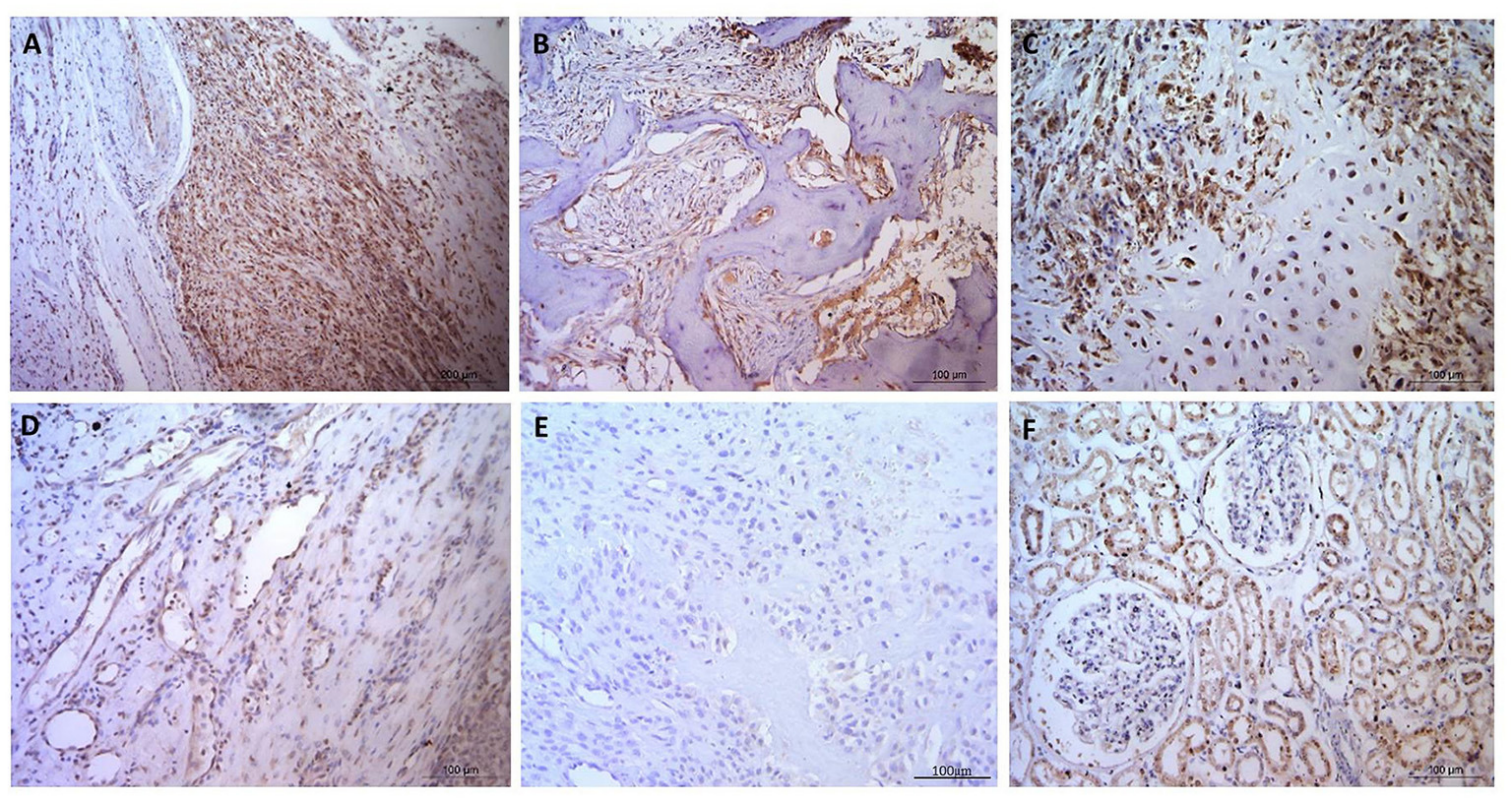
Osteosarcoma GLUT1 immunohistochemical staining. **(A–C)** GLUT1 indicating positive nuclear and cytoplasmic immunostaining, immunopositive blood vessels with more pronounced cytoplasmic staining in the tunica intima in comparison to the tunica media, and negative staining in the osteoid and in **(D)** a tumor-free area. **(E)** Negative control OSA tissue showing no positive immunostaining. **(F)** Positive control canine kidney tissue staining primarily in the tubular epithelial cells. Scale bars represent 100 μm.

Despite GLUT1 staining being observed in every OSA specimen, only half of the specimens stained with GLUT1 antibodies showed individual staining intensity scores of 3, and it was notable that blood vessels frequently exhibited H-score 3 nuclear staining in the tunica intima, whereas generally nuclei in the tunica media exhibited lower H-scores (Figure 1). Cytoplasmic staining produced higher H-scores in the tunica intima in comparison to the tunica media whereas the osteoid matrix did not exhibit immunopositive staining (Figure 2). Generally staining was less pronounced in neoplastic cells in comparison to the endothelium.

### MMP3 H-Score and Expression in OSA

For MMP3 H-scoring (*n* = 51 sections from 12 patients), all specimens showed positive immunostaining and less variation was calculated between patients on the 0–300 score in comparison to GLUT1 and NRF2 H-scoring. In total 8/12 (66.7%) patients had a low average nuclear score, while only 1/12 (8.3%) had a high nuclear score. In addition, 50% (6/12) of the patients had a combination of moderate average cytoplasmic scores and low average nuclear scores. Only 1 (8.3%) patient had a low average cytoplasmic score. Low scores were more likely to be observed in the nucleus, whereas moderate and high scores were more frequently observed in the cytoplasm (*P* < 0.0001). Overall, on the 0–300 scale the cytoplasmic H-scores were higher than those observed in nuclei (*P* = 0.016, Table 1, Figures 1B,D).

The histopathology report showed that staining was diffuse in all cases, with predominantly weak cytoplasmic staining, with some cases showing weak-moderate or moderate cytoplasmic staining (Table 2). The MMP3 nuclear staining was reported as absent or absent-weak in the majority of cases, with some weak and weak-moderate nuclear staining in the remaining cases. In 11 out of the 12 cases, staining was predominant in the cytoplasm, whereas the remaining sample had staining distributed equally between the cytoplasm and nucleus (Table 2).

**Figure 3:**
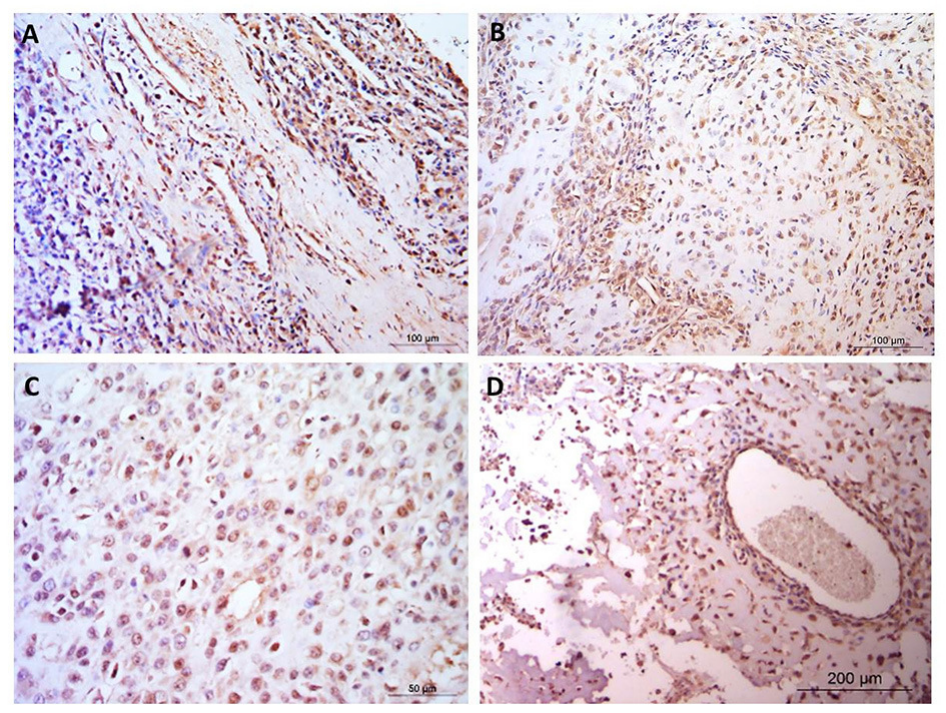
Osteosarcoma immunohistochemical staining for MMP3. **(A–D)** MMP3 positive staining in blood vessels, cytoplasm and nuclei but negative staining in the osteoid, in four canine osteosarcoma samples. Scale bars represent **(A, B)** 100 μm, **(C)** 50 μm, and **(D)** 200 μm.

Staining was observed in the endothelium in all cases and also within the fibroblastic cells present. In addition it was noted that endothelial cell staining intensity was comparable the neoplastic cell staining observed. In contrast, some focal areas vascular/perivascular cells had distinct negative staining which contrasted to neoplastic positive staining observed. In addition connective tissue, muscle and blood vessels predominantly showed weak, diffuse staining and muscle fibers were negative within the nucleus. One case showed rare, weak cytoplasmic staining in the suspected leukocytes. Four patients (33.3%) exhibited positive MMP3 immunostaining in the extracellular matrix, however osteoid staining was not present in any samples (Figure 3).

### NRF2 H-Score and Expression in OSA

NRF2 H-scores (*n* = 51 sections from 10 OSA patients) showed considerable variation between patient averages on the 0-300 scale, but positive immunostaining was observed in all specimens. Some patients (20%, 2/10) demonstrated high average H-scores of both cytoplasmic and nuclear NRF2 staining, some patients demonstrated either high average nuclear staining (20%, 2/10) or high average cytoplasmic staining (10%, 1/10). The remaining 50% (5/10) demonstrated moderate levels of both nuclear and cytoplasmic NRF2 staining. In addition, low and high scores were more likely to be present in the nucleus in comparison to the moderate scores which were more frequent in the cytoplasm (*P* = 0.008). Overall the 0–300 cytoplasmic and nuclear staining H-scores were similar (within 5%, *P* > 0.05), however the range of H-scores was greater in the nuclear staining (Table 1, Figures 1C,E).

The histopathology report showed diffuse staining in 100% of the samples (Table 2). The majority of samples showed absent, weak or weak-moderate cytoplasmic and nuclear staining intensities. In the majority of samples, staining was predominantly observed in the cytoplasm, but in the remaining samples staining was either predominantly nuclear or equally cytoplasmic and nuclear (Table 2).

**Figure 4:**
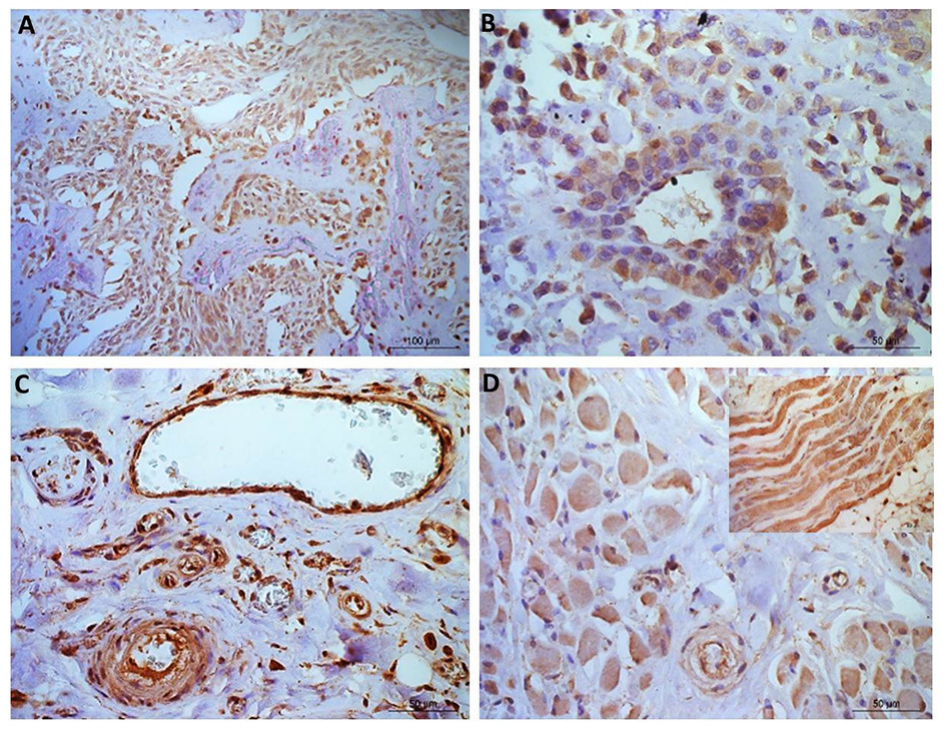
Osteosarcoma immunohistochemical staining for NRF2. **(A, B)** Immunopositive staining in blood vessels, primarily nuclear, in all 10 samples within the tunica intima and in 40% of samples tunica media staining was observed. **(C)** Negative staining in the osteoid (tumor free area). [**(D)**+inset] Muscle tissue showing positive NRF2 staining. Scale bars represent **(A)** 100 μm, **(B–D)** 50 μm.

NRF2 immunopositive staining was observed in every blood vessel, with positive cytoplasmic staining in the tunica intima (all ten samples; 100%), while 8 patients also showed positive nuclear staining in the tunica intima (8/10, 80%; Figure 3). Positive staining was less frequent in the tunica media (4/10 positive cytoplasmic, 3/10 positive nuclear). Muscular tissue was present in 30 of the sections analyzed from across the patients. It was of interest that all 30 sections showed positive NRF2 immunostaining (100%), 6 slides showed heterogeneous immunostaining (20%) in terms of both distribution and stain intensity, while the remainder (80%) showed homogenous staining. Muscle, nerves and connective (adipose/fibrous) tissue presented with diffuse staining which was generally more pronounced than the neoplastic cell population. Where mucosa and inflammation were present, the staining intensity was similar to that noted in neoplastic cells. The osteoid matrix remained immunonegative in all specimens (Figure 4).

## Discussion

In recent years a detailed understanding of the transcriptional heterogeneity and mechanistic processes in human osteosarcoma has been established by the rigorous unbiased transcriptomic analysis of match tumor and non-malignant specimens (36–40). Such knowledge is driving advances in diagnostics and treatment for this disease in man.

Canine OSA remains challenging to treat and carries a poor prognosis due to the very aggressive and metastatic nature of the tumors. Given how common OSA is in dogs and likely genetic contribution to OSA in large breeds, we (16) and others (25, 41–43) have sought to extend understanding of the molecular determinants of OSA in dogs and to compare these results to those obtain from OSA from people.

Here we investigated three cancer promoting proteins that have been shown to be up regulated at the gene level in canine OSA compared to normal bone tissue (16). Indeed the importance of GLUT-1 (37, 44), MMP3 (36, 37) and NRF2 (45) is well established in human OSA. More recently a study used single cell RNAseq to investigate the cellular heterogeneity within human osteosarcoma specimens and identified *MMP-3* as one of the top differentially expressed genes in OSA specimens (40). However little is known about the expression of these in canine OSA tissue.

While glucose is an essential part of cellular metabolism, glucose metabolism is enhanced in malignant cells (46). Glucose transporter member 1 (GLUT1, also known as SLC2A1) is a cell membrane glycoprotein responsible for glucose transport that is widely expressed across cell types and is overexpressed in many cancers (46, 47). The transcription factor hypoxia-inducible factor 1-alpha (HIF-1α) was found to induce GLUT1 thus increasing survival in hypoxic conditions by allowing increased anaerobic glycolysis (48). Additionally, increased GLUT1 expression allows cancer cells to survive low glucose conditions (49), and hypoxic tumor cells are resistant to conventional therapeutics, highlighting the potential of glycolytic inhibitors in osteosarcomas (50). In human OSA, higher expression of *SLCA1I* correlated with a poor prognosis, shorter disease-free interval and increased angiogenesis (47). GLUT1 staining was previously identified in 74% of human OSA specimens and linked with increased tumor volume and metastatic potential, as well as increased recurrence rate (44).

In canine OSA, a study of 44 canine osteosarcoma specimens showed 61% positive GLUT1 staining but no significant correlation was identified between GLUT1 and disease-free interval (51). Interestingly, Petty and colleagues also showed a subset of canine OSA with no or low GLUT1 staining as was seen in human OSA (44). Our findings of GLUT1 in OSA indicated that every specimen had some degree of positive immunostaining, however this varied between patients. Investigations into whether H-scoring differs between tumor grades, type of bone affected/location, sex or other factors still need to be conducted.

GLUT1 staining was observed in every OSA specimen in the present study, and notably blood vessels frequently exhibited H-score 3+ nuclear staining in the tunica intima, and lower H-scores in tunica media nuclei, and cytoplasmic staining in both structures (higher in the tunica intima). GLUT1 immunostaining has previously shown an abundance of the protein in blood vessels within the diaphysis of normal long bones, but not in the metaphysis (52). It has been suggested that osteoblast differentiation is a high-energy demand process, met by upregulation of GLUT1 in bone blood vessels (53). Additionally the importance of GLUT1 in blood vessels has been shown in relation to blood brain barrier function, where the energy demand of the brain during childhood is greater due to the rapidly developing nature of the brain (54). Expressed in both luminal and abluminal endothelial cells within the blood brain barrier (55), haploinsufficiency of SLC2A1 causes GLUT1 deficiency syndrome resulting in delayed development, movement disorders, and seizures (56). These links with high-energy processes could not only explain expression in OSA tissue, but may also highlight GLUT1 as a therapeutic target. The potential of GLUT1 as a therapeutic target has also been demonstrated in human OSA cells, where glycolytic inhibitor sensitized hypoxic cells to chemotherapy (50). Furthermore, increased *SLC1A1* in human OSA microarray datasets has been associated with metastatic tumors and a worse prognostic effect (37). Our findings show that GLUT1 is expressed in canine OSA and confirm the need to investigate the potential of glycolytic inhibitors to increase therapeutic efficacy in both canine and human OSA.

In aggressive tumors, cell invasion and metastases require breakdown of the extracellular matrix [ECM; (57)]. Matrix metalloproteinases (MMPs) are enzymes that degrade the ECM and changes in concentrations of MMPs are important in invasion and metastasis of OSA (58, 59). Metastases in OSA are critical for disease progression and are associated with poor prognosis (60). High expression of MMP3 in many cancers has been associated with poor prognosis (61). In human OSA, MMP3 is highly expressed in OSA tissue in comparison with normal bone (62), and has been shown to be regulated by tumor suppressing microRNAs which are down-regulated in OSA (62, 63). MMP3 may also be associated with OSA metastasis, indeed survival outcomes were improved in patients expressing lower levels of *MMP3* in microarray datasets (37). Complex pathways such as estrogen receptor alpha (ERα) signaling induces FasL transcription in osteoblasts leading to MMP3 expression in these cells, resulting in sFasL production and osteoclast apoptosis (64). Additionally, MMPs are synthesized in stromal cells adjacent to tumor cells (65). Studies in people have also highlighted differentially expressed genes in OSA tissue, including the MMPs and genes which interact with the matrix metalloproteases (36, 59). In the present study, MMP3 staining varied between the canine OSA, including in stromal cells consistent with results in studies of human cancers (65). MMP3 in canine OSA could be used as marker of more invasive and metastatic tumors. Of interest, MMP3 is a druggable target, with a selective inhibitor of MMP3 available (UK370106), but this has not been tested in cancer cells (66). A generic MMP inhibitor Marimastat, showed little promise in clinical trials (67, 68), but has not been tested in OSA patients. More recently, sulfonamide-based inhibitors of MMP3 have also been developed (69). Additionally, MMP3 has been found in extracellular vesicles that were protumorigenic and highly transmissive (70), highlighting another function of MMP3 in metastases and emphasizing it as a potential key therapeutic target in canine OSA.

A feature of OSA and other cancers is chemoresistance. Chemoresistance arises via up-regulation of mechanisms that protect the cell from the impact of chemotherapy. Chemotherapy increases reactive oxygen species (ROS) in cells, which then trigger DNA damage which leads to apoptosis (71, 72). The concentrations of ROS in normal cells are maintained by inducible antioxidants which are regulated by the transcription factor, NRF2 (73). Oncogene- induced NRF2 has been shown to promote ROS detoxification (74) and play a role in tumor progression, invasion, and metastases in many cancers (75). In mice, deletion of NRF2 led to lower bone mineral density and weaker long bones (76). NRF2 has also been implicated in osteoclast activity as when NRF2 was depleted, increased intracellular ROS was observed alongside increased osteoclast numbers, suggesting increased osteoclastic activity with decreased NRF2 (77).

Nuclear staining of NRF2 has been shown in bone metastases of people with OSA (78), and expression of this protein has been associated with poor outcome in OSA patients (45). In our study we observed NRF2 staining in 100% of the canine OSA, however variation in staining intensity was observed between the different patients. This suggests that it has potential as both a prognostic marker and therapeutic target. Knock down of *NFE2L2* in human cancer cells was effective in altering the NFE2L2/NRF2 pathway and improving chemosensitivity (79). Oridonin, a drug isolated from a medicinal herb, has shown potent anti-tumor effects in OSA, by reducing NRF2 and an antioxidant pathway, leading to apoptosis (80). Tanshinone 11A also inhibited OSA growth by targeting AMPk-NRF2 pathway, knockdown of both NFE2L2/NRF2 and AMPK showed same effects as the drug (81). A liposome-based siRNA targeting *NFE2L2*, given in conjunction with cisplatin, improved treatment of OSA (82). These recent developments in pharmacological drugs and RNA interference- based therapies holds promise for treating canine OSA.

Canine OSA is divided into several morphologic subclasses: osteoblastic, fibroblastic, chondroblastic, and teleangectatic (83), however these subclassifications have not yielded significant differences in the prognosis of either human or canine OSA (83–85). In contrast, histologic grading of human tumors, serves as a good indicator for prognosis (85), but is not been widely used as a prognosticator in canine OSA and has failed to be a significant indicator for decreased survival in flat and irregular bones, including the mandible (86–88). However, a mandibular OSA seemed to have a distinctly better clinical outcome than does OSA of other locations (86). Another problematic feature in grading canine OSA is that there are several published histologic grading systems, none of which are universally accepted (89). These difficulties providing a prognosis, make finding suitable markers even more important.

Our results have shown that GLUT1, MMP3 and NRF2 are all present in canine OSA from a number of different anatomical locations including the humerus, scapula, femur, tibia, stifle, carpus and the mandible, maxilla and temporomandibular joint. Previous canine OSA studies have shown that tumor location and mitotic index can be correlated with survival time and disease-free interval (87, 89), therefore understanding expression in the differing locations and mitotic index could be informative. The H-scores of the three proteins varied greatly between individuals in the present study. Although tumor size was not a factor quantified in our clinical samples, it is potentially an area of interest for future work. As larger tumors tend to show more hypoxia and mutagenesis (90), and tumor hypoxia indicated increased expression of GLUT1 in cervical carcinomas (91), this could be an interesting factor to investigate. Higher tumor grades have also been linked to both higher levels of necrosis, and primary lesion location (with appendicular regions often scoring at higher grades) (29); both of these factors are of interest. Therefore larger studies considering multiple factors, such as OSA grade and anatomical location, need to be undertaken and compared to non-tumor tissue, in order to contextualize the complex expression patterns of GLUT1, MMP3 and NRF2. In conclusion, GLUT1, MMP3 and NRF2 are expressed in canine OSA, are good potential candidates for prognostication in OSA and therapeutic targets, and clinical trials using drugs which already target these proteins are encouraged. In addition to understanding canine OSA further, this study also supports spontaneous OSA in dogs as a model system to inform the development of new methods to diagnose and treat OSA in both dogs and people.

## Data Availability Statement

The original contributions presented in the study are included in the article/supplementary material, further inquiries can be directed to the corresponding author/s.

## Ethics Statement

The animal study was reviewed and approved by University of Nottingham School of Veterinary Medicine and Science. Written informed consent was obtained from the owners for the participation of their animals in this study.

## Author Contributions

CR and NM: conceptualization. CR, JC, SS, AA, CB, SB, and NM: data curation. CR, JC, JL-R, SB, and NM: formal analysis. CR, SB, and NM: funding acquisition. CR, JC, JL-R, AH, JJ, SS, AA, CB, AB-R, AR, MD, SB, and NM: investigation. CR, JL-R, CB, SB, and NM: methodology. CR, MD, and NM: project administration. CR, MD, SB, and NM: resources. CR, JR, and NM: supervision. CR, AA, and JL-R: validation. CR, JC, AA, CB, and SB: visualization. CR, JC, AH, JJ, CB, AR, and NM: writing—original draft. CR, JC, JL-R, AH, JJ, SS, AA, CB, AB-R, AR, MD, SB, and NM: writing—review and editing. All authors have read and agreed to the published version of the manuscript.

## Conflict of Interest

The authors declare that the research was conducted in the absence of any commercial or financial relationships that could be construed as a potential conflict of interest.

## Publisher's Note

All claims expressed in this article are solely those of the authors and do not necessarily represent those of their affiliated organizations, or those of the publisher, the editors and the reviewers. Any product that may be evaluated in this article, or claim that may be made by its manufacturer, is not guaranteed or endorsed by the publisher.
